# Factors affecting insomnia in youth college students

**DOI:** 10.1192/j.eurpsy.2026.10740

**Published:** 2026-07-31

**Authors:** B.-H. Yoon, M.-D. Kim, S. Yoon, S.-Y. Lee

**Affiliations:** 1 Psychiatry, Naju National Hospital, Naju; 2 Psychiatry, Jeju National University Hospital, Jeju; 3 Psychiatry, Wonkwang University Hospital, Iksan, Korea, Republic Of

## Abstract

**Introduction:**

Insomnia is one of the most prevalent sleep disorders among young adults, particularly university students, and is associated with impaired daily functioning, psychiatric comorbidities, and increased health risks. Despite the high burden, few studies have examined multidimensional risk factors in Korean college populations.

**Objectives:**

This study aimed to investigate sociodemographic, behavioral, and psychological correlates of insomnia among young college students in Korea.

**Methods:**

A cross-sectional survey was conducted among 5,273 students aged 19–34 years from 14 universities in Jeollanam-do, Korea. Participants completed validated questionnaires including the Korean version of the Insomnia Severity Index (ISI-K), the Center for Epidemiologic Studies Depression Scale (CES-D), and the Generalized Anxiety Disorder-7 (GAD-7). Sociodemographic and lifestyle characteristics such as gender, residential status, socioeconomic perception, smoking, alcohol, caffeine, and smartphone use were also assessed. Group comparisons and multivariate logistic regression were performed to identify independent predictors of insomnia risk, with statistical significance set at p < 0.05.

**Results:**

Overall, 31.1% of participants were classified as being at risk for insomnia (ISI-K ≥ 8). Females had a significantly higher prevalence compared with males (56.8% vs. 43.2%, p = 0.016). Living alone, low perceived socioeconomic status, and lack of close friendships were associated with elevated insomnia risk. Smoking (OR = 1.59, 95% CI: 1.37–1.86, p < 0.001) and alcohol use (OR = 1.23, 95% CI: 1.02–1.49, p = 0.030) emerged as significant behavioral factors. In contrast, caffeine consumption and smartphone use did not show significant associations. Psychological distress was the strongest predictor: depressive symptoms (CES-D ≥ 21) increased insomnia risk more than five-fold (OR = 5.26, 95% CI: 4.22–6.54), and anxiety symptoms (GAD-7 ≥ 10) tripled the risk (OR = 3.03, 95% CI: 2.14–4.29).

**Conclusions:**

This study demonstrates that insomnia in university students is shaped by an interplay of demographic, psychosocial, and mental health factors rather than lifestyle behaviors alone. Depression and anxiety were the most powerful predictors, underscoring the need for early psychological screening and integrated mental health interventions. Public health strategies in Korea should incorporate structured sleep education, counseling services, and policies that strengthen social and economic support systems for young adults.

**Disclosure of Interest:**

None Declared

